# Growth Hormone insensitivity (Laron syndrome): Report of a new family and review of Brazilian patients

**DOI:** 10.1590/1678-4685-GMB-2018-0197

**Published:** 2020-01-20

**Authors:** Thais R. Villela, Bruna L. Freire, Nathalia T. P. Braga, Rodrigo R. Arantes, Mariana F. A. Funari, Jorge A L Alexander, Ivani N. Silva

**Affiliations:** 1 Universidade Federal de Minas Gerais, Faculdade de Medicina, Hospital das Clínicas, Divisão de Endocrinologia Infantil e do Adolescente, Belo Horizonte, MG, Brazil.; 2 Universidade de São Paulo, Hospital das Clínicas da Faculdade de Medicina, Unidade de Endocrinologia Genética, São Paulo, SP, Brazil.; 3 Universidade de São Paulo, Hospital das Clínicas da Faculdade de Medicina, Unidade de Endocrinologia do Desenvolvimento, Laboratório de Hormônios e Genética Molecular, São Paulo, SP, Brazil.

**Keywords:** Laron Syndrome, growth hormone, growth hormone receptor, genetics

## Abstract

Laron’s syndrome (LS) is a rare genetic disorder characterized by insensitivity to growth hormone (GH). Up to the present time, over 70 mutations of GH receptor (GHR) gene have been identified leading to GH/insulin-like growth factor type 1 (IGF1) signaling pathway defect. The number of LS patients worldwide is unknown, as many are probably undiagnosed. We report two sibs from a consanguineous family from Minas Gerais, southeastern Brazil. The parents have three children. The older, a 4-years-old girl was 80.2 cm tall (-5.7 SDS height/age), and the youngest sister, aged 3 years, was 73.2 cm tall (-5.82 SDS height/age). Their clinical and biochemical features are typical of LS patients, such as high serum level of GH and low IGF1 concentrations. A homozygous c.1A>T nucleotide substitution in GHR exon 2 in the probands’ samples was identified. Their parents and healthy sister are heterozygous for the same variant that abolishes the translation initiation codon of GHR. This mutation has not been reported in Brazilian patients and was previously associated with an LS phenotype in a single 29-year-old Spanish man. In addition to this case report, we summarize the main characteristics and molecular data of the 21 LS Brazilian patients who have been published to date.

Laron’s syndrome (LS) is a rare genetic disorder characterized by inability to respond to endogenous or exogenous growth hormone (GH). It is associated with mutations in the GH receptor (GHR; OMIM: *600946), leading to GH/insulin-like growth factor type 1 (IGF1) signaling pathway defect. Currently, instead of being recognized as a single entity, it is accepted as a broad diagnostic category, comprising a range of molecular defects in the GH-IGF1 axis. In most cases, LS is determined by a fully penetrant autosomal recessive mechanism seen more commonly in consanguineous families and, in the vast majority, a molecular defect has been identified involving either homozygous or compound heterozygous mutations. The *GHR* gene is located on the short arm of chromosome 5 and includes 9 coding exons (Fang *et al.*, 2007). Up to the present time, over 70 mutations of GHR gene have been identified including deletions, missense and nonsense point mutations and at splice site. Different phenotypes may occur with the same mutation and within the same family (David *et al.*, 2011). Homozygous mutations in the signal transducer and activator of transcription 5B gene (STAT5B; OMIM: *604260) were also described in patients with GHI (Pugliese-Pires *et al.*, 2010). STAT5B is a critical molecule involved in GHR signal transduction, mediating growth-promoting actions. In addition to its role in growth-promoting actions, STAT5B is also involved in the immune system regulation (Pugliese-Pires *et al.*, 2010).

Patients with LS have characteristic biochemical features, such as high (or normal) serum level of GH and low IGF1 concentration (Laron, 2004; Laron *et al.*, 2012). Clinically it is characterized by dwarfism, obesity, small genitalia in the boys, and severe hypoglycemia. Patients present a typical head configuration, a small face and a protruding forehead, resulting in a saddle nose. Their voices are high-pitched and they are sparse haired. Phenotypically they resembled what was recognized as GH deficiency (Laron and Kauli, 2016). The majority of patients with STAT5B mutations also present severe immune dysregulation and elevated prolactin levels.

The number of known and/or published LS patients is around 350. Most cases have been reported from the Mediterranean region and Southern Ecuador, and a few cases from South America (Laron, 2016; Laron and Kauli, 2016). In Brazil, 19 patients with LS have been reported so far. Most of them carry the E180 splice mutation (c.594A>G, p.V199_M208 del; rs121909360) (Jorge *et al.*, 2005; Goncalves *et al.*, 2014). In this report, two sibs from a consanguineous family, with a mutation in the exon 2 of *GHR*, are described.

A 4-year-old girl (patient 1) was evaluated for short stature. She has presented severe growth retardation since the first postnatal year. She was 80.2 cm tall (-5.7 SDS height/age), had a BMI of 15.4 (-0.2 SDS) and a 2 year bone age. Parents are first cousins with no relevant medical history. They were born in Minas Gerais, southeastern Brazil, and had three children. She is the oldest sibling. Length at birth was 46 cm (-1.7 SDS for age) and weight 3.450 kg (0.5 SDS for age). Clinical examination revealed typical features suggestive of LS: prominent forehead, depressed nasal bridge and high-pitched voice. At her latest visit, she was 9 years old, 105.5 cm tall (-4.95 SDS height/age), 21.1 kg (-2.06 SDS weight/age) and had a BMI of 19.0 (1.01 SDS). The patient started the exchange of deciduous teeth at 8.5 years old. Nowadays she has two permanent teeth and the remaining 22 are deciduous. Delay of dentition is also a LS characteristic(Campbell *et al.*, 2009; Laron and Kauli, 2016). The middle sister was 6 years old, 116.3 cm tall (0.3 SDS) and had no complaints. The 3-year-old youngest sister (patient 2) was 73.2 cm tall (-5.82 SDS height/age), weighed 8.3 kg (-5.92 SDS weight/age) and had a BMI of 15.5 (-0.46 SDS). Length at birth was 46 cm (-1.7 SDS for age) and weight 3400 g (0.4 SDS for age). She also presented typical LS features, including sparse hair and late closure of the fontanelles.

The workup performed for disclosing short stature etiology (blood count, kinetics of iron, urinalysis, renal and hepatic function, lipid metabolism, cortisol, TSH and free T4, acid-base equilibrium, calcium metabolism, sweat test, and antibodies for Celiac Disease) was normal for both patients, except for the GH axis evaluation (Table 1).

**Table 1:** GH axis evaluation of two Brazilian siblings with LS.

Patient	Age at the test	IGF 1 (μg/L)	IGFBP3 (mg/L)	Basal GH (ng/mL)	GH stimulation (ng/mL)
1	4 years old	12.3	0.70	21.5	GH peak>40
		(RV: 49-289)	(RV: 1.0-4.7)		
2	1 year old	79	0.5	11.4	
		(RV: 55-303)	(RV: 0.7– 3.9)		

During follow-up, both patients presented hyperlipidemia. Patient 1 had total cholesterol (TC) = 305 mg/dL, with LDL-C = 242 mg/dL (RV<100 mg/dL) and patient 2 had TC=240 mg/dL and LDL-C=177 mg/dL. After nutritional interventions, slight improvement in cholesterol levels was observed. In their latest visit, patient 1 had TC=243 mg/dL and LDL-C= 157 mg/dL and patient 2 had TC=264 mg/dL and LDL-C=190 mg/dL. Parents had normal cholesterol levels. The neuropsychomotor development of both patients was normal for their age.

Based on clinical suspicions of severe GH insensitivity, genomic DNA was isolated from peripheral blood leukocytes from the two probands. *GHR* exons 2–10 (reference sequence NM_000163.4) were amplified using specific intronic primers to cover the entire coding region (primer sequences and amplification protocols will be sent upon request). PCR products were directly sequenced by the dideoxy chain-termination method and analyzed by an autosequencer. A homozygous c.1A>T nucleotide substitution in GHR exon 2 in the probands samples was identified. Their parents and healthy sister are heterozygous for the same variant. This variant, which abolishes the translation initiation codon of GHR (p.Met1?), is absent in large public data bases (ABraOM: http://abraom.ib.usp.br/ and gnomAD http://gnomad.broadinstitute.org/). It has been previously associated with an LS phenotype in a Spanish patient (Quinteiro *et al.*, 2002) and classified as pathogenic according to ACMG-AMP criteria (Richards *et al.*, 2015).

Growth hormone insensitivity syndrome (GHI) was first described in 1966 by Laron and collaborators (Laron *et al.*, 1966). They reported a Jewish child born in Yemen presenting severe growth deficit. The child had clinical characteristics compatible to GH deficiency, although showing high plasma levels of this hormone. This patient was from a consanguineous family and had two siblings presenting the same pattern. Five older siblings had normal height. After publication of these data, the same group collected another 22 patients from 14 consanguineous families, all coming from the Middle East or Arabian Peninsula and with the same characteristics. Until then, the etiology of short stature was attributed to an inactive GH molecule (Laron *et al.*, 1968).

In 1989, cloning of the GH receptor enabled the identification of partial gene deletion in the GH receptor in two patients (Laron and Kauli, 2016). The first was a 35-year-old male born from related parents of Jewish Iraqi origin. He reached a final height of 128.3 cm (-7.3 SDS for age). The second was a girl sharing the same origin. At 8 years and 10 months her height was 104 cm (-4.2 SDS for age). No serum GH binding protein activity was found in both of them (Godowski *et al.*, 1989).

The total number of patients with GHI worldwide is not known, as many are probably undiagnosed (Laron and Kauli, 2016). It is a rare disorder and 21 Brazilian patients have been reported to date, including the present. Among the reported LS Brazilian patients, 18 have undergone molecular studies. The first two male sibs with GHI reported from Brazil were homozygous for a substitution of T for G at the -1 position of the 3`splice consensus sequence of intron 6 (c.619-1 G>C). Parents were first cousins from Italy (Saldanha and Toledo, 1981; Berg *et al.*, 1993). A third patient carried a homozygous mutation, replacing serine by isoleucine in the codon 244 of exon 7 (p.S244I). Parents are also consanguineous (Jorge *et al.*, 2004). Another patient carried a mutation in homozygous state, adenine duplication at nucleotide 338 (c.338dupA) of the exon 5. This nucleotide alteration causes a premature termination signal at codon 113 (p.Try113*), predicting a truncated GHR. It determines a failure in receptor function caused by the lack of amino acids comprising the transmembrane and intracellular regions of GHR proteins (Diniz *et al.*, 2008). Another 8 patients, from six families, carried a homozygous substitution of guanine by adenine in codon 198 of exon 6 (c.594 A>G), creating an abnormal splice site deleting 8 amino acids from the extracellular domain of GHR (Jorge *et al.*, 2005; Goncalves *et al.*, 2014). This mutation in codon 198 is also known as E180 splice mutation. Four patients with STATB5 mutations, from two different families, have been reported in the South of Brazil. The first two carried a heterozygous missense mutation at exon 5 and both siblings carried a deletion of four nucleotides in exon 5 of STAT5B (Pugliese-Pires *et al.*, 2010). Two siblings from another family, who died of respiratory failure, had their mutations inferred because their parents were heterozygous carriers for *STAT5B* c.424_427del mutation. The prevalence of their *STAT5B* mutation in the South of Brazil was higher than the frequency observed in public databases, supporting the existence of a founder effect (Scalco *et al.*, 2017). Another three patients with dwarfism, high serum levels of GH and low IGF1 concentrations were reported in Brazil, but they hadnt undergone molecular tests (Jorge, 2008).

Finally, in the present paper, we describe two sibs with LS, carrying a homozygous A-T transversion in exon 2. This transversion occurs at the first base pair of the translation initiation codon of the gene. As a result, the methionine marking the starting of the reading frame is replaced by a leucine. Both parents are heterozygous for the same mutation. A summary on the data for the Brazilian patients is shown in Table 2.

**Table 2:** Characteristics of confirmed Brazilian LS patients.

Patient	Consanguinity	Site of mutation	Type of mutation	c.DNA mutation	Amino Acid Change	Main reported Features	Ref.
1	+; Family I	GHR	Splice	Intron 6: c.619-1 G>C		At 13 years old: 87.5cm (-8.5 SDS) and 12.4kg. Several hypoglycemic episodes. Late closure of fontanelles. Trunkal obesity, high-pitched voice, doll-like face, irregular hypoplastic teeth and small external genitalia. Absence of pubertal development.	Saldanha and Toledo, 1981
2	+; Family I	GHR	Splice	Intron 6: c.619-1 G>C		At 8 years old: 76cm (-9.4 SDS) and 10kg. Several hypoglycemic episodes. Late closure of fontanelles. Trunkal obesity, high-pitched voice, doll-like face, irregular hypoplastic teeth, small external genitalia and learning difficulties.	Saldanha and Toledo, 1981
3	+; Family II	GHR	Missense	Exon 7: c.731 G>T	p.S244I	At 15.7 years old: 124cm (-6.1 SDS), 43.4 kg and BA=13.2 years. Weight at birth (full term) = 2 kg (< 3rd centile). GH=12mcg/L, IGF1 <18mcg/L, IGFBP3= 1.1mg/L.	Jorge, *et al.*, 2004
4	?; Adopted; Family III	GHR	Nonsense	Exon 5: c.338 dupA	p.Y113X	At 12 years old: 87.8cm (SDS) and 11.1kg. Length at birth = 44cm, and Weight= 3kg. Facial asymmetry, prominent forehead, depressed nasal bridge, short face, blue sclerae and microstomia. Severe dental crowding and high-pitched voice. Small penis (10^th^ percentile). GH=26 mcg/L, IGF-1=22.5 ng/mL.	Diniz *et al.*, 2008
5	?; Family IV^*^	GHR	Splice	Exon 6: c.594 A>G	p.V199_M206del	At 17.8 years old: 104.3cm (?7.8 SDS); GH =30mcg/L, GH peak =94mcg/L, IGF1 =23mcg/L, IGFBP3= 0.6mg/L.	Jorge *et al.*, 2005
6	+; Family V^*^	GHR	Splice	Exon 6: c.594 A>G	p.V199_M206del	At 8.8 years old: 103.3cm (?5.0 SDS); GH 7.2mcg/L, GH peak 118mcg/L, IGF1 <18mcg/L.	Jorge *et al.*, 2005
7	?; Family VI^*^	GHR	Splice	Exon 6: c.594 A>G	p.V199_M206del	At 11.3 years old: 113.8cm (-4.5 SDS); GH= 3.3mcg/L, GH peak= 36mcg/L, IGF1 <18mcg/L, IGFBP3= 0.6mg/L.	Jorge *et al.*, 2005
8	?; Family VI^*^	GHR	Splice	Exon 6: c.594 A>G	p.V199_M206del	At 6.8 years old: 81cm (-7.6 SDS); IGF1 <18 mcg/L, IGFBP3= 0.4mg/L.	Jorge *et al.*, 2005
9	?; Family VII^*^	GHR	Splice	Exon 6: c.594 A>G	p.V199_M206del	At 19.1 years old: 132cm (-6.4 SDS); GH 1.6mcg/L, GH peak= 38mcg/L, IGF1= 51mcg/L.	Jorge *et al.*, 2005
10	?; Family VII^*^	GHR	Splice	Exon 6: c.594 A>G	p.V199_M206del	At 3.2 years old: 69cm (-7.4 SDS); GH peak =39mcg/L, IGF1 <18mcg/L.	Jorge, *et al.*, 2005
11	?; Family VIII	GHR	Splice	Exon 6: c.594 A>G	p.V199_M206del	No data	Gonçalves *et al.*, 2014
12	?; Family VIII	GHR	Splice	Exon 6: c.594 A>G	p.V199_M206del	No data	Gonçalves *et al.*, 2014
13	+; Family IX	GHR	Missense	Exon 5 (GHR): c.409 G>A	p.317N	At 6 years old: 86cm (SDS), 10kg and BA= 3 years. Length at preterm birth (28 weeks) =39cm and Weight= 1.7kg. Mild dysmorphic features, atopic eczema, interstitial lung disease, hyperprolactinemia. GH peak =20.6mcg/L, IGF1=34ng/mL. Treatment with GH whithout clear improvement.	Pugliese-Pires *et al.*, 2010
		STAT5B	Frameshift	Exon 5 (STAT5B): c.424_427 del	p.L142fs X161		
14	+; Family IX	STAT5B	Frameshift	Exon 5: c.424_427 del	p.L142fs X161	At 2 years old: 76cm (SDS), 7,5kg and BA=6.0 months. Length at preterm birth (33 weeks) = 49cm and Weight= 2.4kg. Prominent forehead, depressed nasal bridge and obesity with centripetal fat distribution. Atopic eczema, thrombocytopenic purpura, interstitial lung disease, hyperprolactinemia.	Pugliese-Pires *et al.*, 2010
						GH peak =14.2mcg/L, IGF1 < 25ng/mL. Treatment with GH and IGF-1 whithout clear improvement	
15	+; Family X	STAT5B	Frameshift	Exon 5: c.424_427 del	p.L142fs X161	Deceased as a consequence of respiratory failure	Scalco *et al.,* 2017
16	+; Family X	STAT5B	Frameshift	Exon 5: c.424_427 del	p.L142fs X161	Deceased as a consequence of respiratory failure	Scalco *et al.,* 2017
17	+; Family XI	GHR	Misstart	Exon 2: c.1A>T	p.M1?	At 4 years old: 80.2cm (??? SDS) and BA=2.0 years. Length at birth = 46cm and Weight= 3.450kg. Prominent forehead, depressed nasal bridge, high-pitched voice and delayed dentition.	Present study
GH peak>40.0mcg/L, IGF1=12.3ng/mL.							
18	+; Family XI	GHR	Misstart	Exon 2: c.1A>T	p.M1?	At 2 years old: 73.2cm (SDS). Length at birth = 46cm and Weight= 3.400kg. Prominent forehead, depressed nasal bridge, high-pitched voice, sparse hair and late closure of fontanelles. GH =11.4mcg/L, IGF1=79ng/mL.	Present study

BA=bone age

* Family IV-VII are proceeding from a small city of the state of Pernambuco, and consanguinity cannot be ruled out.

The mutation found in this new Brazilian family was also reported in a single 29-year-old LS Spanish patient, whose parents of Spanish origin were consanguineous. At the age of 10 he was unsuccessfully treated with GH. He reached a final height of 124.4 cm (-7.5 SDS for height) (Quinteiro *et al.*, 2002).

Research in the field of GHI due to mutations affecting GH action has evolved considerably since the original description of the severe phenotype related to homozygous GHR mutation over 50 years ago. To date over 70 mutations have been reported (David *et al.*, 2011). The most frequent GHR mutation, E180 splice, seems to have originated from a single ancestor and spread to Latin America (Goncalves *et al.*, 2014). The mutation reported here, in this new family, has never been reported in Brazilian patients and could have followed the same pathway. Up to now, no information has shed light on these specific aspects.

In conclusion, we reported a new Brazilian family showing a rare GHR mutation only found previously in a single LS Spanish patient. These findings, according to what has been described above, could be an additional evidence for the possible origin from a single common ancestor of these individuals.
